# Behavioral structure of users in cryptocurrency market

**DOI:** 10.1371/journal.pone.0242600

**Published:** 2021-01-12

**Authors:** Ayana T. Aspembitova, Ling Feng, Lock Yue Chew

**Affiliations:** 1 Division of Physics and Applied Physics, Nanyang Technological University, Singapore, Singapore; 2 Institute of High Performance Computing, Agency for Science, Technology and Research, Singapore, Singapore; 3 Department of Physics, National University of Singapore, Singapore, Singapore; 4 Complexity Institute, Nanyang Technological University, Singapore, Singapore; 5 Data Science and Artificial Intelligence Research Centre, Nanyang Technological University, Singapore, Singapore; Yahoo, SPAIN

## Abstract

Human behavior as they engaged in financial activities is intimately connected to the observed market dynamics. Despite many existing theories and studies on the fundamental motivations of the behavior of humans in financial systems, there is still limited empirical deduction of the behavioral compositions of the financial agents from a detailed market analysis. Blockchain technology has provided an avenue for the latter investigation with its voluminous data and its transparency of financial transactions. It has enabled us to perform empirical inference on the behavioral patterns of users in the market, which we explore in the bitcoin and ethereum cryptocurrency markets. In our study, we first determine various properties of the bitcoin and ethereum users by a temporal complex network analysis. After which, we develop methodology by combining *k*-means clustering and Support Vector Machines to derive behavioral types of users in the two cryptocurrency markets. Interestingly, we found four distinct strategies that are common in both markets: optimists, pessimists, positive traders and negative traders. The composition of user behavior is remarkably different between the bitcoin and ethereum market during periods of local price fluctuations and large systemic events. We observe that bitcoin (ethereum) users tend to take a short-term (long-term) view of the market during the local events. For the large systemic events, ethereum (bitcoin) users are found to consistently display a greater sense of pessimism (optimism) towards the future of the market.

## Introduction

It is well-known that financial systems are complex and their evolution depends heavily on the behavior of their agents (users). This realization can be traced back to the times of Adam Smith in the late 1700s. Subsequently, there are many theories trying to model the complexity of financial systems based on historical patterns (Keynesian economics), with the eventual emergence of the paradigm of “rational expectations” in the twentieth century. Rational expectation assumes that people have access to all the information, act rationally, and adapt fast to new conditions. Theories based on this behavioral assumption, however, are not able to model the real situation observed in markets although they give insights into key aspects of market behavior. It was soon realised that human behavior is more heterogeneous and complex than what efficient market theory has assumed. It spurred the invention of many agent-based models which simulate diverse users (agents) interacting according to a set of prescribed rules. These models are able to explain many stylized facts in financial time series that previous models have failed to reproduce. Although these models perform well in explaining facts observed in financial data, they are based on hypothetical assumptions that a person’s behavior can be subjected to confounding interpretations.

Due to the sensitivity of financial data, there is little opportunity to construct behavioral models on the basis of empirical findings. However, researchers have found ways to gain experimental evidence by conducting laboratory studies [1–3]. The downside of the experimental approach is the limitation of sample size which prevents generalization of its findings to real financial market. The invention of blockchain and cryptocurrencies has overcome this problem by opening up large data-sets of financial transactions for close examination. In addition, it has enabled researchers to study transaction networks, user activities, money flow, etc. from data that is distributed in nodes and servers throughout the world. Several studies have contributed to the understanding of bitcoin network’s structure, evolution [4, 5], and price formation [6]. There is, however, a dearth of research done on the ethereum network. Nonetheless, recent studies [7, 8] have shown similarities of its network properties with that of bitcoin. The study of user behavior in the cryptocurrency market has also mainly been conducted from the point of view of anomaly detection [9–11]. To the best of our knowledge, there are as yet no studies done on the behavioral structure of the users of cryptocurrency market. An understanding on the behavioral structure of cryptocurrency users would allow us to answer questions such as which strategies would the users follow in the cryptocurrency market, and how different or similar they are from those adopted in the behavioral models of other financial markets.

The goal of our research is to first develop the methodology that would allow us to derive the type of strategy employed by the cryptocurrency users from the blockchain data. Next, we investigate into the behavioral structure of the cryptocurrency users and elucidate the number of different strategies exist in the real market. We aim to gain insights into the behavioral composition of these users in the two largest cryptocurrency systems in the market: bitcoin and ethereum. We investigate into the composition of user behavior in response to events that happened at different periods of these cryptocurrency systems: local price fluctuations in bitcoin and ethereum; and shocks in the whole cryptocurrency system termed as Crypto Bubble and Crypto Winter. Our interest is to look at the *persistent* behavioral patterns rather than at the high-frequency strategy switches—the users might change their strategies every day, but we want to look at their overall attitude during these periods. For this purpose, we construct temporal transaction networks of cryptocurrency at an interval of one month for both the bitcoin and ethereum systems, and examine the properties of the constructed networks. Then, we define the set of features that allow us to distinguish strategy types and ascertain their presence for all the nodes in our networks. We implement various machine learning methods to find clusters of users with different behavioral patterns. Overall, it is possible to detect user strategies in cryptocurrency markets and we are able to define four distinct behavioral types universal for both the bitcoin and ethereum systems. We found that during local price fluctuations, ethereum shows more stable behavioral composition compared to bitcoin where changes in price evolution tend to change a user’s behavior. Our analysis also shows that systemic events change people’s behavior in both systems, but quite differently—there was no big change in ethereum with slight increase of number of pessimistic users, while bitcoin users appeared to be more optimistic.

The organization of our paper is as follows. In section 2, we provide an overview on the current state of research relating to the understanding of trading behavior in financial markets in general and in cryptocurrency markets in particular. We then review research on the application of machine learning techniques to blockchain data. In section 3, we introduce our dataset and explain how we perform feature selection and extraction in our paper. In section 4, we describe our developed methodology for defining strategies from the data set. In addition, we show the implementation of this methodology to extract behavioral patterns and discuss our obtained results. Finally, we conclude our paper and propose potential future directions for our research.

## Related work

There are plenty of research conducted on behavioral types in financial markets and various models have been proposed. A thorough review of existing agent-based models has been done in the thesis of Feng [12] and the review of Iori [13], where they showed the evolution of agent-based modelling in finance. LeBaron [14] has provided a systematic review of artificial financial markets by classifying them into “few-type” and “many-type” models.

On the other hand, research on strategies and users behavior in cryptocurrency markets are few. Cocco [15, 16] has proposed an agent-based model to explain price movements in bitcoin. They assumed that there are two types of behavior in the bitcoin system: chartists and random traders. The authors then prescribed behavioral rules to the agents according to their type and observed how they affect the market price of bitcoin.

As for experimental research to understand the behavior of users of the cryptocurrency system, interesting work has been done by Krafft [17]. These researchers have conducted online experiments to study how users are susceptible to peer influence in cryptocurrency markets. By placing experimental orders in the Cryptsy exchange and then observing users’ behavior afterwards, they assessed the strength of peer influence on the users. This study has shed light on the understanding of causal impact of individual opinion in large cryptocurrency markets.

The use of machine learning methods in blockchain and cryptocurrency data sets is not new and has already been implemented for various purposes. The most popular task is to use machine learning to detect anomalous user behavior. The authors in [9] have analysed bitcoin transaction network data for the four years (2009-2013) with the goal of detecting suspicious users. They used Local Outlier Factor (LOF) to first detect outliers in the dataset, and then employed k-means clustering to calculate the relative distances between the cluster centroids and the detected outliers. This enabled them to estimate the performance of LOF—if the computed distance is small, LOF has performed poorly. Overall, the authors were able to detect anomalous transactions using this approach. Another study on the detection of anomalous user behavior has been conducted by Monamo [10]. They used trimmed k-means clustering to detect outliers, i.e. data points that are farthest away from the cluster centroids. In [11], the authors trained a supervised machine learning algorithm to predict the category of the unidentified users. Identified users (a sample of 957 of the 385 million transactions) were used as a training set for the Gradient Boosting algorithm, and classifiers were built to differentiate users among 12 categories—exchange, mining pool, personal wallet, scam, darknet, ransomware, hosted wallet, gambling, mixing, stolen coins, merchant services and others. Interestingly, they are able to predict the category that the user belongs to with an accuracy of 80%.

Overall, the detection of user types in cryptocurrency systems is mainly to address the security and privacy issues. Furthermore, most of recent research focuses on the bitcoin transaction network, with less work being performed to understand the cryptocurrency system as a whole [18]. So far, lots of work have been done to understand the behavior of financial markets’ participants both at the theoretical and experimental levels [13, 17, 19, 20]. These approaches have been successfully applied to cryptocurrency markets as well [15, 16]. However, there is still a lack of studies that derive users behavior in financial markets from empirical evidences. The successful implementation of machine learning methods in identifying anomalies and user categories has inspired us to employ them for the identification of behavioral patterns in cryptocurrency system which would contribute to the understanding of human behavior in financial markets.

## Data and methods

### Data description

Bitcoin transaction data was extracted from the full Bitcoin blockchain starting from the genesis block (dated 3 January 2009) up to block 560,000 (dated 25 January 2019). It was then processed with BitIodine software [21] which implements clustering of addresses into those hypothetically belonging to the same user based on two heuristics: (1) several addresses transacting to one account are considered to belong to one user; and (2) the address of a transaction which appears to be a “change” transaction is considered to belong to the sender of the transaction. “Change” transaction is the unspent output that Bitcoin protocol forces to use as an input in the other transaction. Based on the processed data [22], the temporal network of interactions of bitcoin users was estimated. Since the clustering algorithm is heuristics-based, it does not guarantee that all the wallets in the network are clustered to corresponding users. Therefore, we might expect a certain fraction of non- or poorly clustered wallets, but still this algorithm results in a significant improvement of network representation of financial interactions in bitcoin. Example of the bitcoin dataset is demontrated in Table 1:

**Table 1:** An example of bitcoin’s dataset obtained using BitIodine software [21].

Sender key	Receiver key	Date	amount
501194	2645	20101105221244	0.90
1EHwci1gVKrs	1986	20101105221244	0.10
834628	834630	20100718123114	2.00
834628	C8x2hqqgE2b	20100718123114	0.05
713610	5188	20110218223432	50.00

The data contains information on the users’ ID (both sender and receiver), exact date and time of the transaction, and the amount of transaction in bitcoins. Every wallet is reflected as a hash in the blockchain. For users with only one wallet, their user ID is the same as the hash of their wallet. In the case when a user has more than one wallet, all his/her wallets are clustered into one unit and a random number is assigned to the cluster. The user ID in this case is the ID number of the wallet cluster.

The difference between the blockchains of ethereum and bitcoin is that the balances in ethereum’s nodes are stored directly in an account. Therefore, when obtaining data from ethereum’s blockchain, there is no need to perform the de-anonymization procedure that is necessary for bitcoin. In our research, we have used the processed ethereum dataset from [23] and example is shown in the Table 2.

**Table 2:** An example of ethereum’s dataset obtained from [23].

Sender key	Receiver key	Date	amount
0xea674fdde7	0x52bc44d537	20180105221244	5.70
0x209c4784ab	0x2a65aca4d5	20180105221244	0.30
0x61c808d82a	0x7ed1e469fc	20180018123114	1.00
0x209c4784ab	0x52bc44d537	20180018123114	03.05
0xb2930b358f	0xabbb6bebfa	20180018223432	2.00

Similar to bitcoin, the data contains information on users’ ID (both sender and receiver), exact data and time of the transaction, and the amount of transaction in ethereum. Since de-anonymization and wallet clustering is not required for ethereum’s data, user IDs are the original hashes of the users’ wallets.

### Network construction and properties

Our interest is to elucidate the behavioral composition of the users at different periods of the bitcoin and ethereum systems. For this, we first define periods of distinct price behavior, i.e. price increase, stable price, and price decrease, separately for each currency. To define the trend of price movement, the average relative daily return and the total relative return in one month for both cryptocurrencies are calculated. Table 3 shows the chosen periods with the return values.

**Table 3:** Periods when bitcoin and ethereum systems show significant and non-significant price changes.

Period	Average return	Total return	Trend
BTC Oct 2015	1.2%	28%	Increase
BTC Dec 2014	-0.1%	-17%	Decrease
BTC Apr 2016	0%	0.06%	Stable
ETH May 2017	4%	202%	Increase
ETH Jul 2017	-0.5%	-30%	Decrease
ETH Apr 2017	1%	5.2%	Stable

The average and total returns are calculated for each period, and based on the evaluated value, we define the trend of the price (price increase, price decrease, or stable price).

Next, we analyze user composition in both bitcoin and ethereum during the occurrence of extreme events that affect the entire cryptocurrency system: (a) December 2017—January 2018 (aka Crypto Bubble), (b) the period after this event, and (c) the shock event at the end of 2018 known as Crypto Winter.

We construct a temporal, weighted, and directed network for each of the defined periods where each link (*i*, *j*, *w*, *t*) is a transaction between two nodes (users) *i* and *j* at time *t* with the amount of coins *w*. The main properties of each network are shown in Table 4.

**Table 4:** Network properties calculated for bitcoin (BTC) and ethereum (ETH) networks for each period under analysis.

Period	*V* number	*E* number	Average Degree	Clustering	Assortativity
BTC Oct 2015	2,606,248	6,448,463	2.47	0.12	-0.07
BTC Dec 2014	1,636,554	4,173,671	2.55	0.12	-0.064
BTC Apr 2016	4,473,678	10,048,780	2.25	0.1	-0.056
ETH Apr 2017	364,504	725,205	1.99	0.104	-0.115
ETH May 2017	774,119	1,567,020	2.02	0.096	-0.105
ETH Jul 2017	1,387,519	2,890,970	2.08	0.091	-0.169
BTC Dec 2017	12,593,945	22,716,559	1.8	0.101	-0.1
BTC Feb 2018	6,194,508	10,777,423	1.74	0.05	-0.08
BTC Nov 2018	8,629,863	14,184,283	1.64	0.04	-0.04
ETH Jan 2018	8,788,002	18,748,342	2.13	0.065	-0.055
ETH Mar 2018	4,278,371	7,656,868	1.79	0.097	-0.085
ETH Dec 2018	3,055,070	5,430,566	1.78	0.153	-0.122

Overall, we can see that all the constructed networks are large, with millions of nodes (*V*) and links (*E*) for most cases. The networks are also quite sparse with an average degree of about 2 and a small clustering coefficient. Both bitcoin and ethereum networks are slightly disassortative, with a coefficient of around −0.1.

### Feature extraction and analysis

From the constructed networks, we calculate properties (features) of each user. Our goal is to yield the various strategies employed in the cryptocurrency market. According to the literature [12–14], agents in the financial markets tend to adopt one of the following strategies: buy, sell, trade or hold. To see if this behavioral composition holds in the cryptocurrency market, we need the following information from each user: (i) frequency and amount of transactions, (ii) total accumulated/spent coins, and (iii) number of outgoing and incoming transactions. Based on the constructed networks with properties shown in Table 4, it is possible to obtain the required information.

The following are features that we shall use to define the type of strategy employed in the cryptocurrency market:

- Total degree of a node *i* at time interval *t*. (This feature corresponds to the total number of interactions of node *i* at time interval *t*, which reflects the transaction frequency of a user.)
- In-degree of a node *i* at time interval *t*. (This feature corresponds to the total number of interactions at time interval *t* with the node being a receiver of the transaction.)
- Out-degree of a node *i* at time interval *t*. (This feature corresponds to the total number of interactions at time interval *t* with the node being a sender of the transaction. These two features: in- and out-degrees of a node, help us understand how active a node is in sending/receiving coins.)
- Outgoing value. (This is the total amount of eth/btc sent in the time interval *t*. It is the sum of the weights *w* of a node *i* when it is a sender of the transaction.)
- Incoming value. (This is the total amount of eth/btc received in the time interval *t*. It is the sum of the weights *w* of a node *i* when it is a receiver of the transaction.)
- Total balance. (This is the net number of coins in the account balance of node *i* in the time interval *t*. These three features: incoming value, outgoing value, and balance of a node show the wealth of a node and its preference to accumulate or spend coins.)
- Total transacted value. (This is the sum of outgoing and incoming values of node *i* in the time interval *t*.)

**Fig 1:**
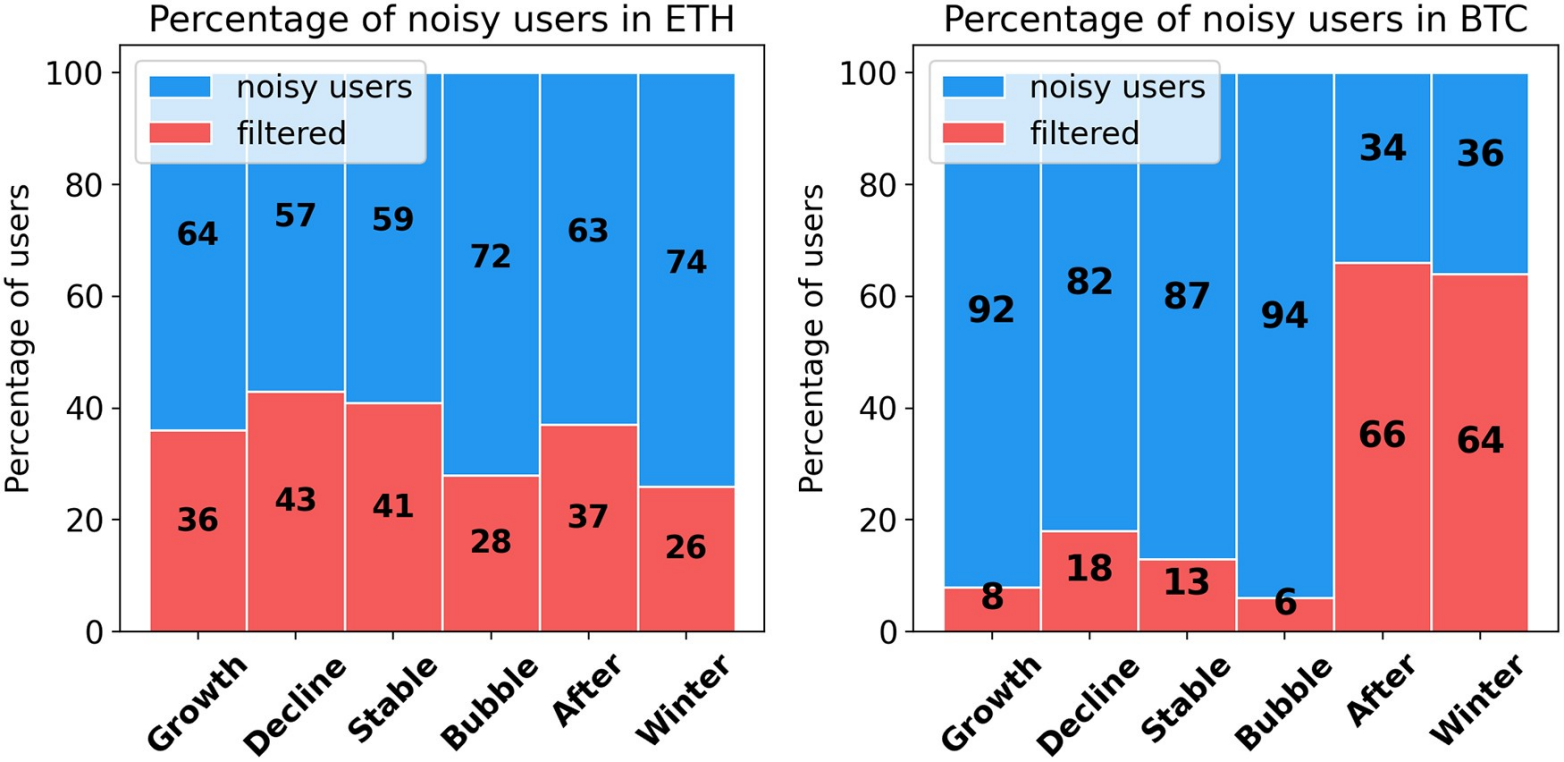
Proportion of “noisy” users in transaction networks. More than 50% of users in each period of ETH have degree less than 2, small transaction value and appear only once. In bitcoin vast majority are “noisy” users but surprisingly, in the period of Crypto Winter and after Crypto Bubble the proportion of “noisy”users is only 36-38%. “Noisy” users are removed for the further analysis.

Due to the novel nature of cryptocurrencies, there are many users both in bitcoin and ethereum that are experimenting with system by placing random orders and leaving the system quickly. We consider these users as noise and omit them from the analysis, since they do not contribute to the overall understanding of market behavioral structure. Moreover, they cause confusion to our analysis by the machine learning algorithms as we try to define the existing behavioural pattern in the market. We define “noisy” user as follows—total degree for whole transaction history is less than 2, and traded value is less than 1% of all transacted values at that time step. However, it should be noted that not all “noisy” users in bitcoin can be considered as experimenting users—some of them might be the consequence of BitIodine clustering limitations, as discussed in the Section. Overall, the percentage of “noisy” users during different periods shown in Fig 1:

Then features are calculated for all users under analysis in each network (see Table 4). In [4, 5] it has been shown that degree and wealth in the bitcoin network are power-law distributed with the exponent around 2. We found that indeed, in, out and total degree are power-law distributed with the exponent around 2-2.3, while in, out and total value are also power-law distributed with the exponent around 1.7-1.9. Heavy tailed degree and wealth distributions in various financial markets is well-known fact and has been extensively researched [24–27]. Cryptocurrency market also shows this property similar to other markets.

We then select features for the machine learning model from those listed above. Overall, unsupervised feature selection methods can be categorized as filter and wrapper approaches [28]. Filter approach selects the most relevant features based on certain criteria (correlation, entropy etc.), while wrapper approach first defines subsets of features and then evaluates them based on the result of the certain clustering algorithm. In our research we first calculate correlation between various features, then define a few set of features for further analysis. The final choice of the most optimal feature set will be based on the clustering result.

**Fig 2:**
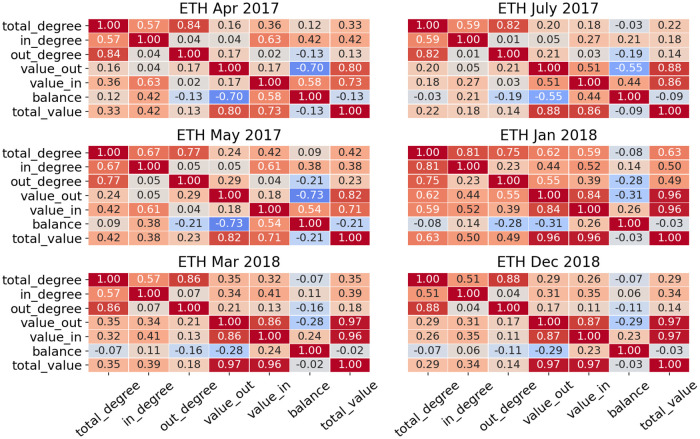
Correlation between all features in ETH are shown—It can be seen that total degree and total value are redundant features as they are always highly correlated with others. In later periods, value in and value out also show very high correlation.

**Fig 3:**
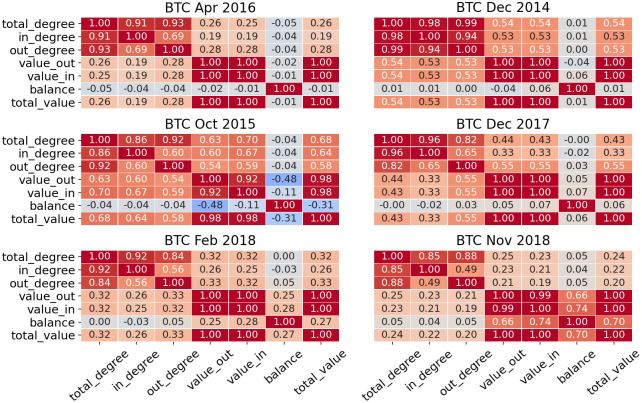
Correlation between all features in BTC are shown—It can be seen that in, out and total degrees are always highly correlated, same as in, out and total value.

Figs 2 and 3 show correlations between features for different periods in bitcoin and ethereum:

After understanding relationships between users’ properties, we define sets of features for each cryptocurrency—*Set 1* includes all features listed above, *Set 2* omits total degree and total value as they showed the highest correlation with other features, *Set 3* includes only features with low correlation, this set is different for etherem (in degree, out degree, total value and balance) and bitcoin (total degree, total value and balance). Later we will use all sets of features and show which set provides better clustering.

## Model for behavioral classification

The detection of behavioral types of users in cryptocurrency system can be considered a clustering problem. By having a set of properties of all users, we can treat these properties as features in a machine learning method and cluster the users with similar behavioral traits in one single group.

### Review of methods

There are many unsupervised clustering algorithms that can be broadly divided into two categories: hierarchical and partitional techniques [29]. Hierarchical clustering produces a dendrogram (a hierarchy of nested clusters) by iteratively merging smaller clusters to larger ones [29, 30]. Although this method provides detailed information on how clusters are related to each other, final output on structural information cannot be clearly visualised when performed on a large number of points. This can result in a wrong interpretation of patterns similarity in dataset. In the study of [31] the performance of various unsupervised clustering algorithms was evaluated—it has been found that *k*-means clustering outperforms other partitional methods like DBSCAN [32, 33] or OPTICS [34] in both one and multi-dimensional feature sets. The authors have also found that spectral methods have higher accuracy than *k*-means clustering when there are more than 10 feature sets. However, with a lesser number of features, their performance was found to be compatible. Therefore, we have decided to use *k*-means clustering because this method has both methodological and computational advantages.

*K*-means algorithm is a partitional squared-error clustering method. It is broadly used for unsupervised clustering due to its computational effectiveness and easy implementation. The method comprises the following steps [35]:

1. Initialize the cluster centroids *μ*_1_, *μ*_2_, …*μ*_*k*_.
2. Segment the data into *k* groups. Assign each data point to the closest centroid and change the centroid to the average of its assigned points so that the distortion function:
  $$\begin{array}{c} J\left(c,\mu \right)=\sum_{i=1}^{m}\left|\right|x^{\left(i\right)}-\mu_{c^{\left(i\right)}}{\left|\right|}^{2} \end{array}$$ (1)
  converges. Note that *x*^(*i*)^ is one of the *m* data points and *μ*_*c*^(*i*)^_ is the cluster centroid assigned to the *i*-th data point, i.e. *c*^(*i*)^ ∈ {1, 2, …, *k*}.

Despite the efficiency of *k*-means clustering, there is a preliminary step where the optimal number of clusters has to first be carefully identified. Various statistical methods offer different ways to calculate this number from the dataset [36–38]. We use the elbow method in the next section to determine the optimal number of clusters for the bitcoin and ethereum cryptocurrency systems.

Support Vector Machines (SVM) is a supervised machine learning method that has gone from being largely unnoticed [39] to a famous method [40]. It is widely applied in the field of digital recognition [41], computer vision [42], and text classification [43]. SVM shows comparable results with neural-network-based algorithms when applied to classification and pattern recognition problems [44]. When used for classification purposes, SVM divides the training dataset with the hyper-plane which is the most distant from the data points. Assume that we have a training labelled dataset

$$\begin{array}{c} S=\left(x^{1},y^{1}\right),\ldots \left(x^{m},y^{m}\right) \end{array}$$ (2)

of size *m*, where **x**^*i*^ is the data point and *y*^*i*^ is its label. The SVM algorithm finds a hyper-plane (**w**, *b*) such that *γ* in the following equation is maximised:

$$\begin{array}{c} \gamma =\underset{i}{\text{min}}\;y^{i}\left\{\left\langle w,\phi \left(x^{i}\right)\right\rangle -b\right\}\;. \end{array}$$ (3)

Note that the quantity {〈**w**, *ϕ*(**x**^*i*^)〉 − *b*} corresponds to the distance between the point **x**^*i*^ and the decision boundary, and when multiplied by the label *y*^*i*^ it gives a positive value for correct classifications and a negative for wrong ones. Minimum of this quantity over the whole data set is positive if data is linearly separable, and *γ* is called the margin. When data is not linearly separable, SVM can be improved with kernels that realize the non-linear mapping to the feature space.

Originally, SVM was designed for binary classification. It has now been extended to cases where there is more than two classes by considering the multi-class problem as a series of binary ones (one-against-one, or one-against-all strategies) [45]. In section, we will show how these methods are used to identify user groups with different strategies within the cryptocurrency systems.

### Methodology

In consequence of the power-law distribution of features, the preliminary step is to normalize and scale the features for proper performance of the machine learning methods. Otherwise, the common machine learning algorithms can lead to biased results due to the data in the fat tail of the distributions. Normalization was performed by taking the logarithm of the original value of the feature: *x*′ = *log*(*x*).

Since our dataset is unlabelled, we do not have information on the existing strategies employed in the cryptocurrency markets. Hence, we use *k*-means clustering to cluster users into groups. But before using *k*-means clustering, we need to identify the optimal number of clusters. We use the elbow method [36] for this purpose and calculate the number of clusters for all datasets and all feature sets. After clustering data using *k*-means we examine the distribution of the original properties of each group of users and analyse how distinct they are from those in the other groups. Based on that, in section we define rules for each cluster and provide the characteristic descriptions of their properties.

We then validate statistically how well our data points fit into each cluster by calculating their silhouette score [38] for all feature sets and all periods as follows:

$$\begin{array}{c} s\left(i\right)=\frac{b(i)-a(i)}{max(a(i),b(i))}\;, \end{array}$$ (4)

where *a*(*i*) is the mean distance between *i* and all other data points in the same cluster, while *b*(*i*) is the smallest mean distance from *i* to all points in the other clusters that *i* does not belong to.

Then we use more advanced technique of Support Vector Machine to cluster users more precisely according to the defined rules.

Overall, our algorithm is as follows:

1. Use elbow method for all feature sets to find the optimal number of clusters
2. Perform *k*-means clustering on the unlabelled feature matrices *A*_*c*_ and obtain the vector of labels *V*_*c*_:
  $$\begin{array}{c} A_{c}=[\begin{array}{cccc} a_{11} & a_{12} & \ldots & a_{1j}\\ a_{21} & a_{22} & \ldots & a_{2j}\\ \ldots & \ldots & \ldots & \ldots \\ a_{n1} & a_{n2} & \ldots & a_{nj} \end{array}]\;,\;\;\;\;V_{c}=\{\begin{array}{c} v_{1}\\ v_{2}\\ \ldots \\ v_{n} \end{array}\}\;. \end{array}$$
  Note that *A*_*c*_ is the feature set for the calculation of the optimal number of clusters for the division of users into distinct groups. Also, the size of *A*_*c*_ is *n* × 7 for Set 1, *n* × 5 for Set 2 and *n* × 4 (ETH) *n* × 3 (BTC) for Set 3, where *n* is the number of nodes (users) and the columns correspond to the defined features. *V*_*c*_ gives the set of labels to the *n* nodes (users) based on the clustering.
3. Analytical validation—examine clusters qualitatively and find the distinct properties in each group of clustered users.
4. Perform statistical validation of clusters by calculating the silhouette score.
5. According to the distinct properties in clusters, define a rule that majority of data points follow in cluster. Adjust *A*_*c*_ and *V*_*c*_ accordingly by removing data points that do not follow the rule.
6. Use adjusted *A*_*c*_ and *V*_*c*_ as a training set for the SVM model.
7. Use the trained SVM model to label the sets of features *A*_1_, *A*_2_, …*A*_*m*_ from other datasets. Each matrix *A*_1_, *A*_2_, …*A*_*m*_ represents feature sets in each period shown in Table 4.

**Fig 4:**
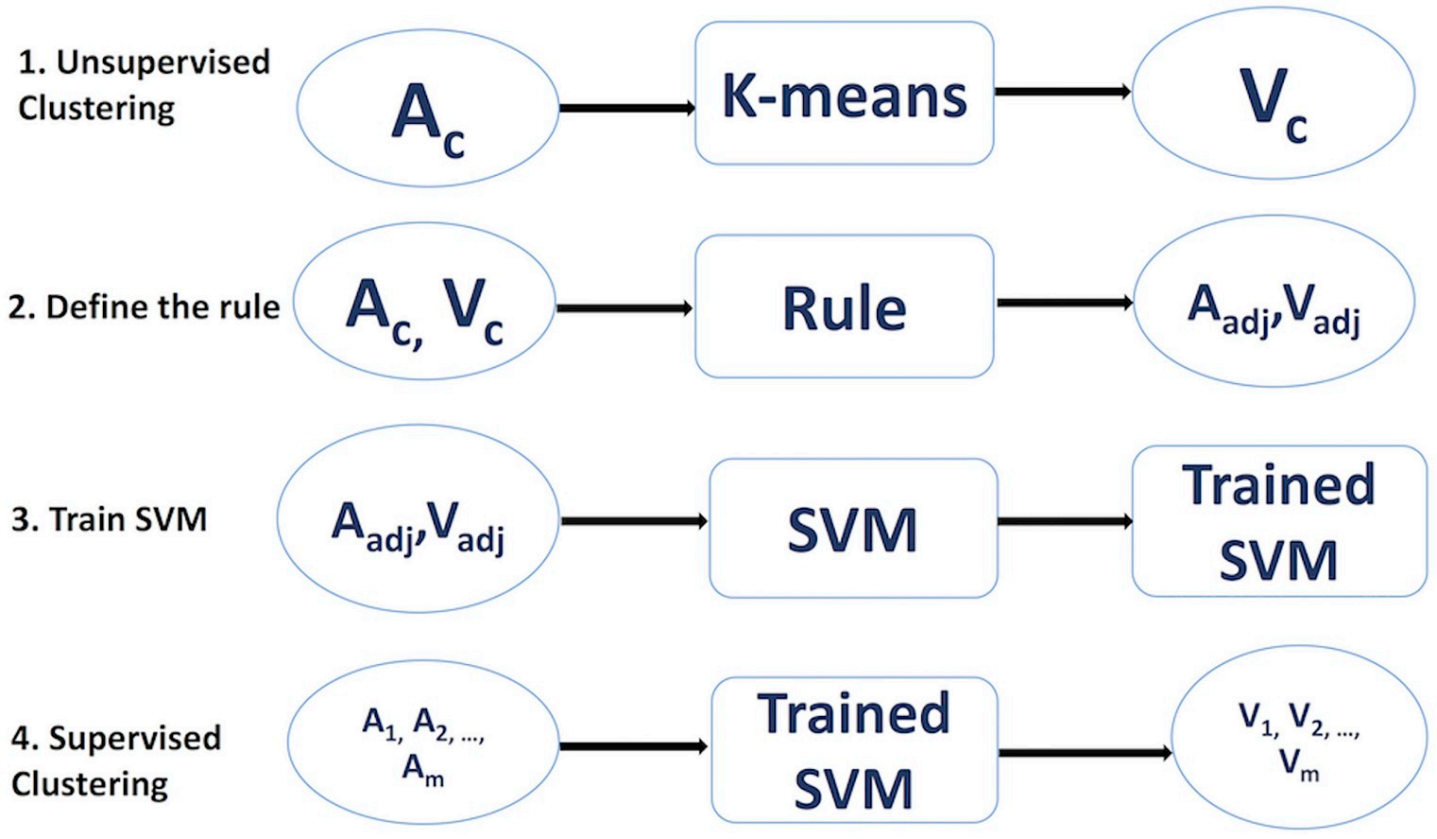
The algorithmic steps to classify users into behavioral groups. Due to the complexity of the feature sets, we found that solely applying unsupervised method is insufficient. Our approach is to first group the set of features *A*_*c*_ that *k*-means clustering has good performance. From which, we obtain the set of labels *V*_*c*_. Subsequently, we use the clustered output as training data for the more advanced supervised algorithm SVM. The final step is to employ the trained SVM model as classifier for the rest of the feature sets *A*_1_, *A*_2_, …*A*_*m*_ to obtain their corresponding labels *V*_1_, *V*_2_, …*V*_*m*_.

Fig 4 illustrates the algorithm described above.

In section, we will discuss in greater detail the results obtained in each step of our algorithm. In summary, our algorithm has allowed us to perform user classification more precisely and it has also increased the speed of the clustering process.

## Results and discussion

**Fig 5:**
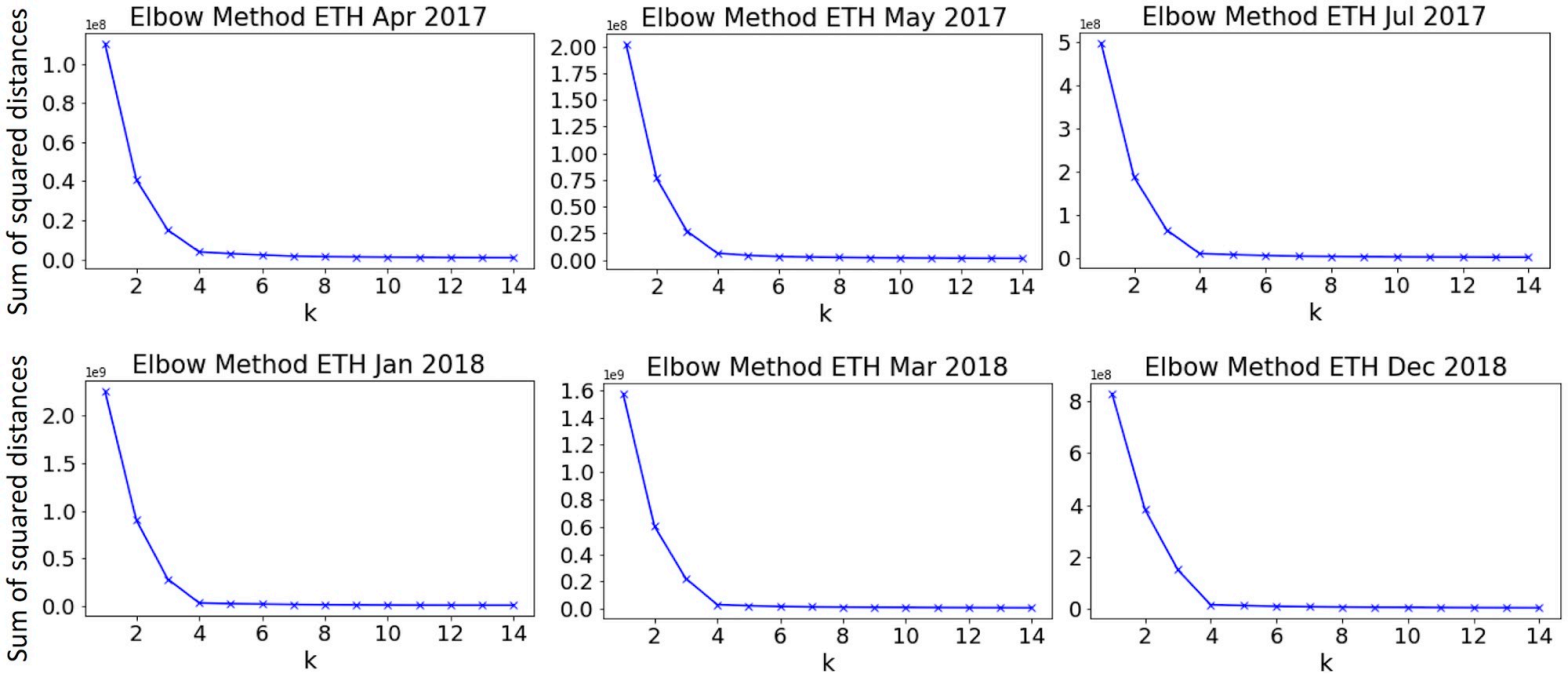
Number of clusters in ETH data found using the elbow method—for all periods, 4 appeared to be the optimal.

**Fig 6:**
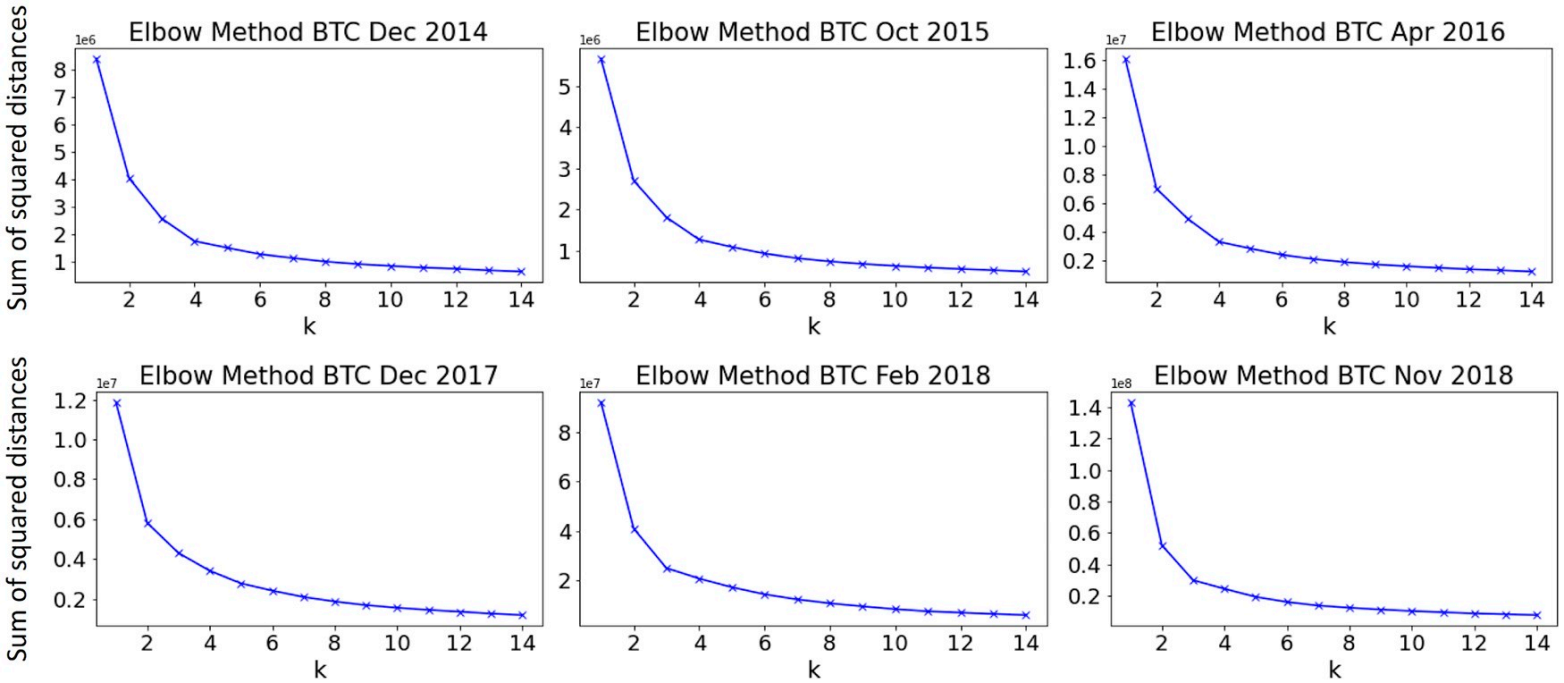
Number of clusters in BTC data using the elbow method—for local events, optimal number is 4. However, for global events there is no “sharp” elbow.

First, we explore how many clusters can be distinguished in dataset—for this purpose, we use the elbow method for all defined feature sets. For ethereum, it has been found that Set 1 and Set 2 produce sharp elbow at 4 clusters (Fig 5, while Set 3 has failed to find optimal *k*. For bitcoin, the best results were obtained using Set 2 and 3 (Fig 6, however for periods of global events, there is no distinct optimal number of clusters regardless of feature set used).

*K*-means clustering with *k* = 4 then allows us to obtain the vector of labels *V*_*c*_ for feature matrices *A*_*c*_ where defining the optimal number of clusters was possible. To understand better about each cluster, we examine the behavior of users in each group and analyse their distinctive features. In order to do this properly, we map the obtained label vector *V*_*c*_ to the original feature matrix *A*_*c*_ before the process of normalization and the scaling of feature values. The Table 5 shows the main characteristic properties for each group that are universal for both bitcoin and ethereum. Note that the negative balance displayed in the table should not be interpreted literally. This is because blockchain technology does not allow users to double-spend or overdraw. A negative balance in our results implies that during the period under consideration, outgoing transactions exceed the incoming ones. Since we are not aware of the initial balance of the users, we cannot estimate exactly the amount each user holds at the moment. Therefore, in our data it is displayed as negative balances.

**Table 5:** Distinct properties of the majority of nodes in each cluster.

	In degree *k*_*in*_	Out degree *k*_*out*_	In value *v*_*in*_	Out value *v*_*out*_	Balance *b*
Group 1	*k*_*in*_ > 0	*k*_*out*_ = 0	*v*_*in*_ > 0	*v*_*out*_ = 0	*b* > 0
Group 2	*k*_*in*_ = 0	*k*_*out*_ > 0	*v*_*in*_ = 0	*v*_*out*_ > 0	*b* < 0
Group 3	*k*_*in*_ > 0	*k*_*out*_ > 0	*v*_*in*_ > 0	*v*_*out*_ > 0	*b* > 0
Group 4	*k*_*in*_ > 0	*k*_*out*_ > 0	*v*_*in*_ > 0	*v*_*out*_ > 0	*b* < 0

Based on their behavioral strategies as shown in Table 5, we name each group as follows:

- Optimists (Group 1)—Users who invest in currency. Their persistent strategy for a period of one month is to buy and accumulate coins.
- Pessimists (Group 2)—Users who sell the currency. They do not buy coins and their balance is negative at the period under consideration.
- Positive Traders (Group 3)—Users who alternate between buy and sell, but have positive balance that shows their preference to accumulate coins.
- Negative Traders (Group 4)—Users who alternate between buy and sell, but have negative balance that demonstrates their pessimistic attitude.

**Fig 7:**
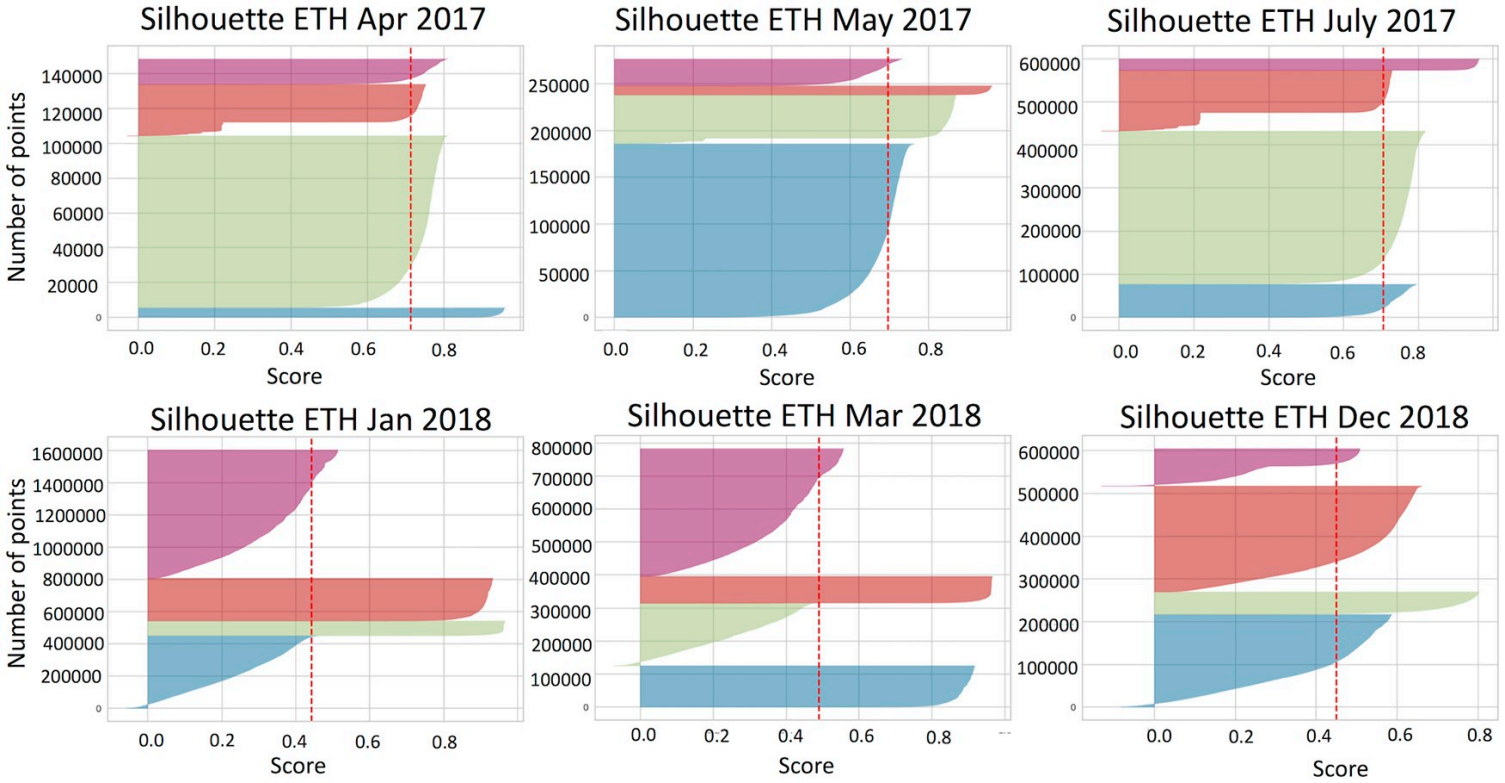
Silhouette coefficient calculated for filtered ethereum data set. For local periods all data points have positive scores, while small number of data points for global events are not well matched with their cluster (have negative score).

**Fig 8:**
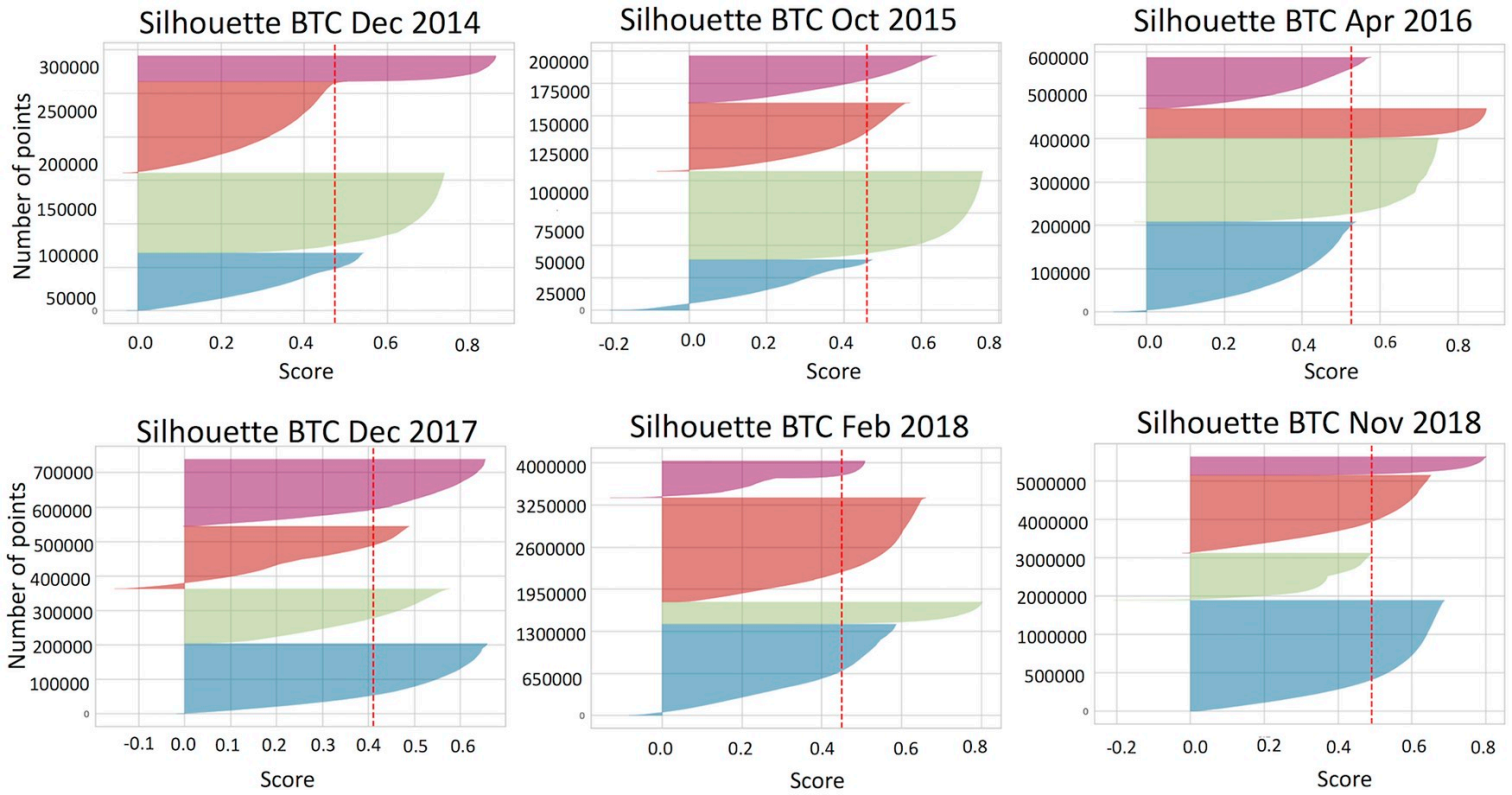
Silhouette coefficient calculated for filtered bitcoin data set. In all periods we see amount of data points with negative score and average silhouette coefficient for all is 0.4.

We next statistically validate if the data is well clustered by calculating the silhouette score for each data point based on Eq (4). Figs 7 and 8 demonstrate silhouette scores calculated for filtered bitcoin and ethereum datasets.

We have found that there are data points in each cluster that have negative silhouette score. These users appeared to have different properties than it is shown in Table 5 and we consider them as misclassified points. Table 6 shows the percentage of misclassified points in each period for various feature sets. Although the silhouette analysis for bitcoin global events shows that most of data points have positive score, users in each cluster do not share similar properties. Therefore, we consider this case as fail for *k*-means to define meaningful clusters.

**Table 6:** The percentage of misclassified points shown in comparison with various feature sets used.

	Set 1	Set 2	Set 3
Local ETH	≈ 0.001%	≈ 0.001%	fail
Global ETH	0.05 − 0.1%	0.012 − 0.016%	fail
Local BTC	fail	0.6 − 7%	3 − 12%
Global BTC	fail	fail	fail

Overall, feature set 2 shows the best performance.

We take the properties listed in Table 5 as an ideal case (rule) for defining the behavioral clusters in cryptocurrency system. Then we use the labelled datasets *A*_*c*_ and *V*_*c*_ (all labelled sets for local periods were merged to increase training set for better performance) and adjust them by removing misclassified points. The method of SVM is then used by treating adjusted *A*_*c*_ and *V*_*c*_ as a training set, the linear kernel for SVM was used and the *C* parameter is equal to 1. We classify datasets for global events using trained SVM and compare properties of users in each group with the ideal case (rule) shown in the Table 5. All points for both bitcoin and ethereum global events were classified properly according to the rule. We would like to mention on the variability among users in the same group, as well as the variability of group properties across different periods. Note that the rules described in Table 5 aim to classify users with certain behaviour (attitude), while the users can be of any type—ranging from independent users to large entities. Therefore, properties (e.g. balance, degree) may vary from very small to very large value. Group properties are also observed to vary across different periods. Although the general rules described in Table 5 continue to hold true for all groups across all periods, the average values of those properties (as well as minimum and maximum) may vary. There are also users with slightly negative (or slightly positive balance) that are classified by our defined rule as members of group 4 (or group 3), although their properties are not much different. We have checked on the number of these users with the slightly negative (or positive) balance (balance that is under the first percentile of all balances in the system at that period). We found that there is a small percentage of ETH users (less than 0.5%) in group 3 whose balance is slightly positive. The number of users with slightly negative balance in group 4 is about 1- 7%. For BTC the number of such users are also less than 0.5% for group 3 and from 6-15% for group 4.

**Fig 9:**
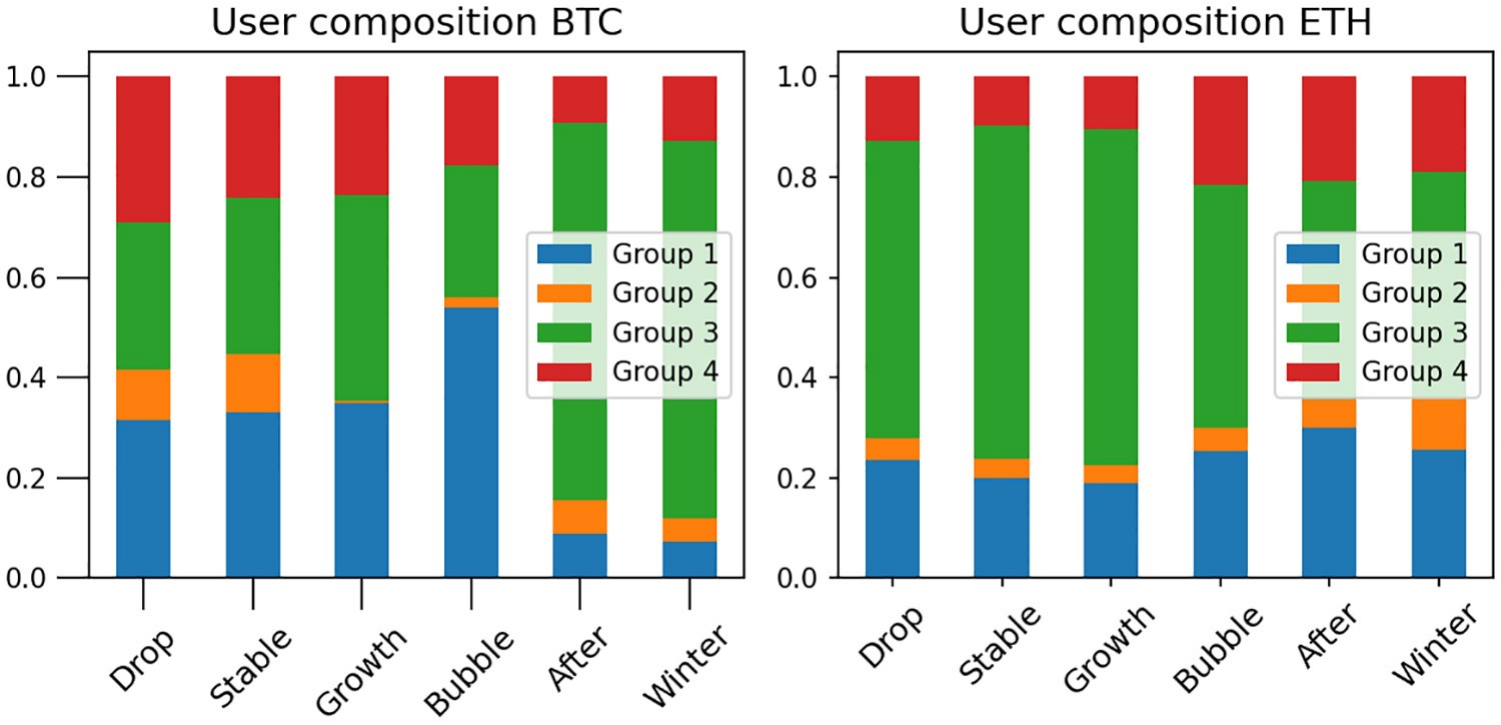
The percentage of users in different groups in the in bitcoin and ethereum during different periods of systems’ evolution.

We then look at the evolution of behavioral types in the cryptocurrency system at different periods: Fig 9 shows the evolution of users behavior in bitcoin and ethereum. Overall, it can be seen that the user composition is more stable in the ethereum system, with the decreased number of positive traders during the shock events—20%-40% smaller comparing with the periods of local events. Consequently, populations of negative traders, pessimists and optimists increased (50%, 10%-80% and 20%-30% relatively). As for bitcoin, people’s behavior is more volatile depending on the price movement. Price decline and stable price periods show the similar behavioral composition, but the growth of price leads to the change in users behaviour—there are no pessimists and number of positive traders increased up to 35%, while number of optimists and negative traders remains the same. As for the systematic events, bitcoin’s users behaviour changes dramatically during the Crypto Bubble with an increase of optimists’ population up to 45%, comparing with the local events. Periods after Bubble and Crypto Winter show very different behavioral composition with the majority of users (up to 70% from total number) being a positive traders. The difference between the two dominant cryptocurrencies could be due to their distinctive nature. Although both are considered ‘currencies’ by many, ethereum has direct utility as ‘gas’ payment that enables computation of smart contracts. This is in contrary to bitcoin, which is mostly considered as a store of value (and sometimes ledger) that many people tend to profit upon its price fluctuations, contributing to the change in user composition during periods of price increase and decrease. Systemic events affect users’ strategies in both currencies compared with the local events, however their strategy choices were quite different. Users in bitcoin appeared to be more optimistic during the Crypto Bubble. The increased number of users in group 3 during the Crypto Winter and after Bubble period might also mean the lack of persistency in optimistic and pessimistic strategies—people switch strategies often and our algorithm averages them out as a group of traders.

## Conclusions and future work

In conclusion, we have constructed empirical human behavioral analysis in financial system during different periods of its evolution. Our developed methodology to classify different strategies that exist in the cryptocurrency market, using a combination of unsupervised machine learning method (*k*-means clustering) and supervised learning method (SVM), has allowed us to derive distinct and robust clusters of users having different behavior. This methodology has been applied to the two largest cryptocurrencies—bitcoin and ethereum, during periods of local price changes and also during large systemic events. Our obtained results show that there are four distinct behavioral types in the cryptocurrency systems: optimists, pessimists, positive traders and negative traders. We have analysed the behavioral composition of bitcoin and ethereum users during the periods under consideration and we found ethereum users’ behavior to be more stable. We infer this behavior to result from the long-term view that the ethereum users have on the market relative to that of the bitcoin users. As for systemic events, users’ behavior changes in both currencies with very different strategy preferences.

Although both bitcoin and ethereum are digital tokens that serve as decentralised currency based on blockchain technology, there are crucial differences between them. While bitcoin has positioned itself as an alternative monetary system in the financial market, ethereum has mostly focused on monetising smart contracts. Also, being the first cryptocurrency, bitcoin has been widely used for speculative purposes. These traits are reflected in the user composition as shown in Fig 9, where the behavior of ethereum users is observed to be more stable as these users are more optimistic of the market. In contrast, the behavior of the bitcoin users tend to fluctuate according to the trend of the market, with a loss of optimism when the market goes down.

By establishing a basic understanding of users strategies in the cryptocurrency financial market, our research can be extended in different directions for the future. This includes improving current agent-based models or constructing new ones to yield the relationship between price movement and people’s behavior based on empirical evidence. Specifically, more work can be done on studying switching mechanisms between different strategies among users—it is assumed that some properties of financial time series such as power-law tails of returns and volatility clustering arise from behavioral switching of market participants [46, 47]. Now, when we are able to identify behavioral groups, it becomes possible to observe the evolution of participants’ behavior and to empirically derive relations with the market effects.
